# Digital inequality, faculty communication, and remote learning experiences during the COVID-19 pandemic: A survey of U.S. undergraduates

**DOI:** 10.1371/journal.pone.0246641

**Published:** 2021-02-10

**Authors:** Vikki S. Katz, Amy B. Jordan, Katherine Ognyanova

**Affiliations:** 1 Department of Communication, Rutgers, The State University of New Jersey, New Brunswick, New Jersey, United States of America; 2 Department of Journalism & Media Studies, Rutgers, The State University of New Jersey, New Brunswick, New Jersey, United States of America; Indiana University Bloomington, UNITED STATES

## Abstract

**Aims:**

The COVID-19 pandemic forced closure of most U.S. university campuses in March 2020, obliging millions of students to finish their semesters via remote learning. This study examines whether and how students’ prior and current experiences of digital inequality—defined as constrained access to the internet and internet-connecting devices—were associated with their remote learning experiences.

**Method:**

An anonymous, online survey of 2,913 undergraduate college students from 30 U.S. universities completing their spring term remotely was conducted between April and May 2020. Hypothesis testing utilized a structural equation model with cluster-bootstrapped standard errors and p-values, to account for students being clustered by university.

**Results:**

Findings revealed that students’ challenges with internet connectivity and digital devices during remote learning were associated with lower remote learning proficiency (RLP). Difficulty communicating with professors and teaching assistants was also associated with lower RLP. Prior experience with online coursework was associated with higher RLP, and digital inequality challenges during the year prior to the pandemic with lower RLP. Moreover, students who reported greater financial hardship since the start of the pandemic experienced significantly more connectivity, device, and faculty communication challenges during remote learning, and had significantly lower RLP.

**Conclusions:**

Many students will continue to learn remotely in some form until the pandemic recedes. We identify key factors associated with students’ remote learning proficiency: (1) consistent, high-speed internet connectivity and functioning devices to connect to it, and (2) the ability to relate to and communicate easily with professors and teaching assistants. This study identifies potential barriers to effective remote learning, as well as possible opportunities to improve students’ experiences.

## Introduction

In March of 2020, the COVID-19 pandemic forced the sudden closure of college campuses across the United States. Within a fortnight, millions of college students were required to finish their semesters online, via remote learning modalities. This study presents results from a survey of 2,913 U.S. undergraduates about their remote learning experiences during the spring 2020 term. We examine whether and how prior and current experiences of *digital inequality*—defined as constrained access to the internet and internet-connecting devices—were associated with remote learning experiences reported by students attending a broad array of U.S. colleges and universities.

We rapidly developed and deployed an online survey to capture the extraordinary, unanticipated learning experience that the pandemic necessitated, as it was unfolding. The study aim is to identify what modifications in students’ learning conditions could have meaningful, positive effects on their remote learning experiences, given that many universities will continue with remote learning in some form until the pandemic recedes. In the sections that follow, we summarize the key aspects of extant research that inform our approach and analyses.

### Digital inequality among U.S. undergraduates

The pivot to remote learning in the spring of 2020 quickly brought digital inequality to the forefront for undergraduate college students. Students who had depended on campus WiFi and university devices suddenly lost those supports when campuses shut down. While we know of no systematic research on digital inequality among undergraduates who returned to their childhood homes to complete their terms remotely, one in three K-12 students experienced digital inequalities that hampered their remote learning in the spring [1]. It is likely that a considerable number of undergraduates from lower-income families experienced those same challenges at the household level, as has been reported anecdotally [2]. Lower-income households often experience disrupted or slowed connectivity stemming from an inability to pay monthly bills consistently or to regularly update their technology [3].

The most common term used to describe digital inequality is “digital divide.” The binary that the term conjures, of digital “haves” and “have-nots” within a population, belies the current reality. Rates of internet access and device ownership are near-saturation in the U.S., particularly among young adults and families with school-age children. By 2016, the Pew Research Center reported that 97% of young adults had internet access and 98% owned a smartphone [4]. A year prior, a nationally representative survey of lower-income families with school-age children reported that 95% had some form of internet access and a device that connected to it [3]. These findings clearly show that binary measures of internet access/no access and digital device ownership/non-ownership are no longer adequately sensitive measures of digital inequality.

As a response to this need for more nuanced metrics for how young people actually experience digital inequality, Katz and colleagues developed measures to evaluate the extent to which individuals and families are “under-connected,” meaning that their internet and devices do not permit them to be as connected as they would like to be [5–7]. In contrast to the dichotomous framing of “digital divide,” being under-connected occurs along a continuum. Their measures capture specific, multi-dimensional ways in which people report being under-connected, including: interruptions in internet access due to unpaid bills in the prior 12 months; internet or digital devices that are too slow or otherwise insufficient for their needs; and needing to share devices among family members, resulting in less time online than they need [5, 7]. Furthermore, Katz and Rideout find relying on a smartphone for connectivity (i.e., being without consistent access to a laptop or desktop computer) is a form of being under-connected; mobile-only connectors go online less often, and for a narrower set of purposes, than those with regular computer access [3].

Where individuals and families would place themselves on the under-connected continuum also varies over time. Gonzales’ research with low-income adults reinforces the necessity of considering digital inequality as a state of “dependable instability,” rather than a time-limited experience [8]. That is, there are times when people with limited financial means can afford to pay for internet connectivity, but also times when they have to prioritize other expenditures. Dependable instability underscores the need to account for variation over prior months or the past year, rather than relying only on the precise moment when participants respond to a cross-sectional survey [3, 8]. Thus, accurately capturing who is under-connected requires measuring multiple dimensions of the experience and measuring changes in those dimensions over time.

Research on undergraduates’ experiences of being under-connected is rare. In large part, this is because, under normal (non-pandemic) circumstances, on-campus resources help to mitigate digital inequality by providing students with WiFi access and devices in campus libraries and computer labs. Gonzales and colleagues have been at the forefront of establishing how digital inequality nonetheless affects undergraduates’ experiences, prior to the pandemic. At a large, public U.S. university, they found that students reported near-universal ownership of cellphones and laptops, consistent with national estimates [9]. However, they also found that nearly one in five students reported “technological maintenance” issues, such as malfunctioning laptops or broken smartphones. Gonzales and colleagues show that delays in resolving those issues, which are time-consuming and adversely affect students’ ability to keep up with their coursework, are more common among lower-income students [9].

### Digital inequality and undergraduates’ online learning experiences

To this point, we have overviewed digital inequality literature that centers on inequity in digital *access*; specifically, access to fully functional digital devices and to high-speed, consistent internet. As the field of digital inequality studies has developed and evolved along with the digital communication technologies that these scholars examine, access is now considered to be the first, and most fundamental, of three, interlocked levels of digital inequality. First-level digital inequality refers to unequal *access* to the internet and digital devices; second-level digital inequality to discrepancies in digital *skills and engagement*; and third-level, to differential *outcomes* of one’s efforts to use digital access and skills to achieve a goal [5, 10–13].

An expansive body of research shows how unequal digital access (first-level digital inequality) affects individuals’ likelihood of developing the necessary skills to fully engage in digital environments (second-level digital inequality). For example, children and adults who have daily internet access are more likely to develop capabilities to successfully locate online information and assess its quality, as well as to engage in digital content production (as opposed to consumption, which requires a much more limited digital skillset) [3, 5]. Consistent with those more general findings, Correa reports a significant association between a personal computer, digital skills, and digital engagement among undergraduates when controlling for all other factors [14].

#### Second-level digital inequality and undergraduate remote learning

Prior research on second-level digital inequality reveals that undergraduates enter college with highly variable levels of digital knowledge and skills [14, 15]. Hargittai and Micheli recently synthesized the extant evidence on second-level digital inequality among undergraduates and young adults. The digital skillsets they highlight as most important for this age group include: (1) awareness of the capabilities of digital technologies, such as how to adjust settings and use platforms to their full capacities; (2) how to use digital technologies to communicate with others; (3) how to participate in digital environments through content creation, and (4) how to seek help from others, ranging from social support to troubleshooting digital issues [16].

The design of a remediation course for first-year college students from rural, Appalachian communities in the U.S. invokes all four skillsets highlighted by Hargittai and Micheli [16]. Welser and colleagues found that prior constraints in digital access and opportunities for skills development (e.g., limited access to high school computing classes) placed these students at a significant comparative disadvantage for college success [17]. Their intervention randomly assigned 373 students in a freshman course to two groups. The treatment group participated in a learning community that introduced students to the capabilities of digital learning platforms (e.g., Google Classroom), including how to adjust settings to track deadlines and feedback. They also scaffolded collaborative skill development and assistance-seeking with other students. Students assigned to the control group took the introductory course with no additional activities. Welser and colleagues report significant increases in students’ self-reported digital skills in the treatment group [17].

Undergraduates who rate their digital skillsets as proficient are more likely to engage in complex digital activities than those who do not, according to Correa [14]. Her survey of undergraduates examined how digital experience, self-reported skills, and motivation were associated with students’ likelihood of digital participation. Correa differentiates between digital skills and what she calls “perceived competence,” self-reported by students, regarding their abilities to successfully participate in online content creation. She concludes: “when perceived competence regarding content creation was included in the analysis, general online skills were not important anymore. This result suggests that competence perceptions regarding specific tasks may override the influence of actual skills in those tasks” [14].

Correa’s conclusion conjures a partial resemblance to the extant literature on self-efficacy in general, and in online learning environments more specifically. As defined by Bandura, self-efficacy is a person’s perception that they can complete a specific task, and their expectation that their efforts will result in favorable outcomes [18]. Self-efficacy has been adapted to study online undergraduate learning environments and to identify the factors associated with students’ success in those learning modalities. Zimmerman and Kulijowich find that higher scores on their online learning self-efficacy scale are positively correlated with the number of online courses a student has completed, their perceptions of their technology skills, and their likelihood of enrolling in future online courses [19]. A separate study by Zimmerman found a negative relationship between the scale and successful completion of an online summer statistics class, suggesting that some students’ self-reports of online learning efficacy are overly confident [20]. Online learning self-efficacy has also been associated with students having greater interest in resolving technology- and learning-related challenges, and feelings of pride [21].

We apply online learning research to our current inquiry with caution, however. Research on online undergraduate learning prior to the pandemic was focused, by definition, on students who chose to take some or all their classes online, with faculty who had chosen, and were prepared, to teach online. The sudden shift to remote learning prompted by COVID-19 forced all students into online learning modalities, regardless of their motivation, digital skills, or experiences of under-connectedness. Those students were also being instructed by faculty who, by and large, had never taught online before, and were therefore also learning as they went along [22]. For these reasons, the applicability of online learning research to remote learning should be cautiously interpreted—particularly in the early months of the pandemic, before faculty and students developed greater competence in remote learning environments.

#### Student-instructor communication during remote learning

When considering how communication between undergraduates and their instructors affected the early weeks and months of remote learning, the second and fourth digital skillsets outlined by Hargittai and Micheli are most relevant: how to use digital technologies to communicate with others, and how to seek help from others [16]. While seeking assistance from instructors does not imply digital skills during non-pandemic semesters, it does during remote learning when all communication occurs via a technological conduit of some kind.

What makes communicating with professors and other instructors (such as teaching assistants) different from the other digital skills that students had to rapidly develop, or adapt, in remote learning is that the success of such efforts is only partially within student control. That is, because student-instructor communication is a bidirectional, asymmetrical interaction in which students are the less powerful interlocutor, this form of communication is distinctive from students’ efforts to communicate with or seek assistance from peers in digital spaces. Furthermore, faculty were also in unfamiliar learning environments in those early weeks and months. The result, in many cases, was changing due dates, requirements, and learning formats [23]. The degree to which faculty communicated clearly with students and maintained clear expectations, predictable deadlines, and consistent learning formats, would also have directly affected how confident students could feel in their capabilities to manage their new learning conditions.

Loepp’s small, but multi-wave, survey of 100 students at a public, midwestern U.S. university during the spring 2020 term reports three main findings that support treating faculty communication as a precursor to students’ developing the necessary skills to competently manage remote learning. Loepp concludes that several efforts are essential for students to develop confidence and persistence in remote learning: (a) regular and clear faculty communication about the course and informal student welfare check-ins; (b) demonstration of compassion to student challenges, including poor internet connectivity and non-functional devices; and (c) utilization of learning platforms in a clear and consistent way that enables students to master them [24].

#### Third-level digital inequality and undergraduates’ online learning experiences

While prior research clearly establishes that digital *access* (first-level digital inequality) and digital *skills* (second-level digital inequality) are linked, it is not yet fully clear how unequal digital access and skills are tied to *outcomes*. Third-level digital inequality is a more recent theoretical construction than first- and second-level digital inequality, but nonetheless a crucial emerging area of study. This is the level that most clearly reveals how digital inequality maps onto more entrenched social inequalities, by tracing how access and skills actually translate into meaningful changes in people’s lives. Helsper and van Deursen have shown how digital access and skills can support material improvements for adults by, for example, securing a new or better job as a result of successfully engaging online resources [13, 25].

There have not been similarly systematic studies linking college students’ digital access and skills to tangible outcomes, such as matriculating into graduate programs or securing jobs after graduation. To date, cross-sectional studies have associated first or second-level digital inequality to interim outcomes. For example, Reisdorf and colleagues found that students at a large, public U.S. university who did not have a laptop during their freshman year had a lower first-year Grade Point Average (GPA), after controlling for other factors [26]. Gonzales and colleagues made a similar connection between technological maintenance issues and end-of-term GPAs [9]. While these findings are suggestive, they are not true third-level digital inequality outcomes like rates of college graduation, for example, would be.

Our study does not include considerations of third-level digital inequality, since the timing of our survey was intended to capture a unique and extraordinary learning experience as it was unfolding, not its outcomes. The question of how remote learning affects undergraduate outcomes will be a crucial area for future study, as the longer-term consequences of the pandemic become clearer in time.

In sum, the goal of this study is to identify key factors associated with undergraduates’ self-reports of *remote learning proficiency* (RLP) during the early weeks of the COVID-19 pandemic in the U.S. We examine how first-level digital inequality reported when the spring term pivoted to remote instruction (i.e., having been under-connected in the prior year) was associated with students’ self-reports of first-level digital inequality *during* remote learning (i.e., connectivity challenges and device challenges), and with students’ abilities to communicate with the instructors teaching their courses (i.e., communication challenges).

**Fig 1:**
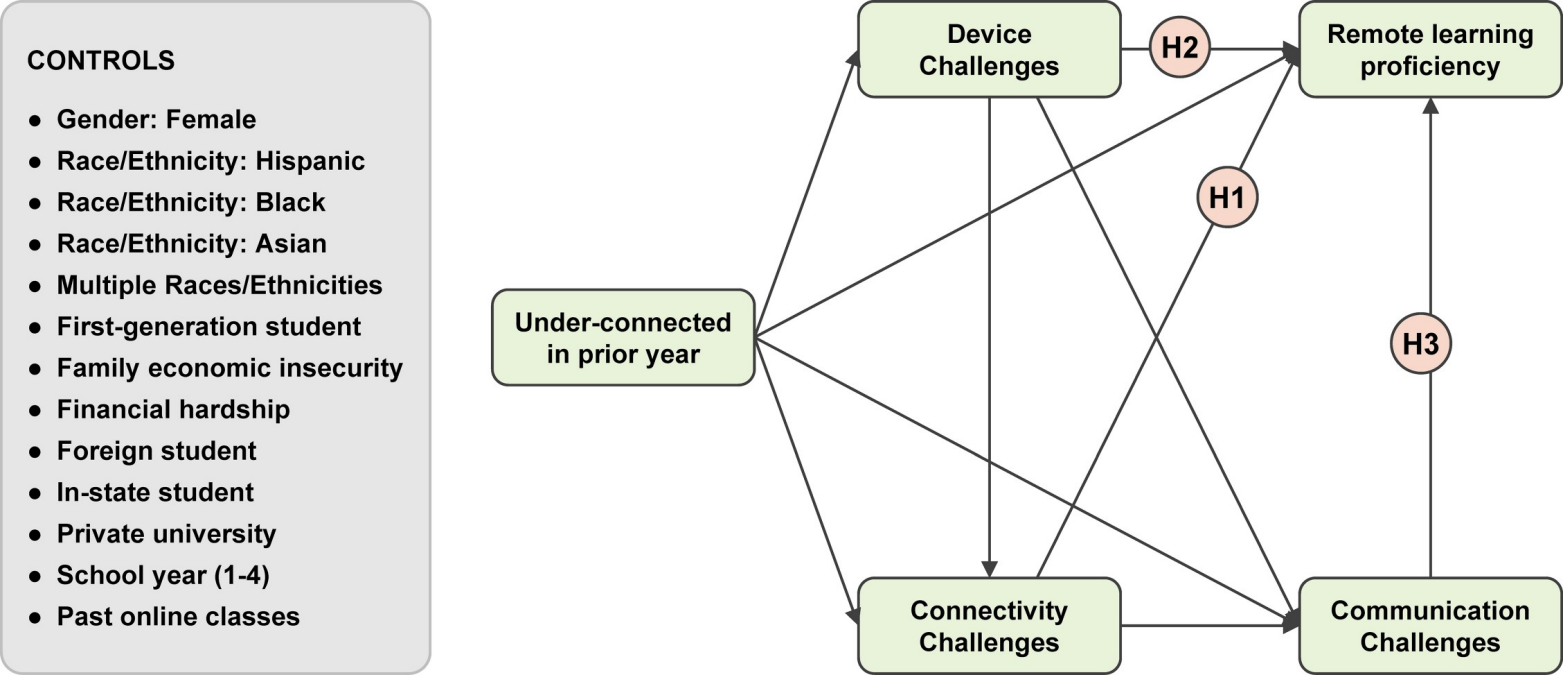
Theoretical model of remote learning challenges and proficiency.

We present and test a theoretical model (see Fig 1) of how these factors are directly and indirectly associated with each other and with students’ RLP, which we consider to be a pandemic-specific manifestation of the differentiated digital skills and engagement that constitute second-level digital inequality.

## Hypotheses

We predict that:

**H1:** Students who report more connectivity challenges during remote learning will feel less proficient as remote learners, compared with students who report fewer connectivity challenges.
**H2:** Students who report more device challenges in remote learning will feel less proficient as remote learners, compared with students who report fewer device challenges.
**H3:** Students who report more challenges communicating with their professors and teaching assistants will feel less proficient as remote learners, compared with students who report fewer communication challenges.

Informed by extant findings on the importance of past technology experiences, we explore whether having been under-connected in the prior year or having taken online courses prior to the pandemic affect students’ remote learning proficiency:

**RQ1:** How are students’ past experiences with technology (i.e., having previously taken online courses, and having been under-connected in the prior year) associated with their remote learning proficiency (RLP)?
  Finally, since digital inequality has consistently been associated with financial challenges in prior research, we examine whether students’ financial circumstances during the early phase of the pandemic are associated with RLP:
**RQ2:** Are students’ assessments of their own and their families’ current financial in/security associated with their remote learning proficiency and challenges with connectivity, devices, and faculty communication?

## Method

This study draws on an online survey of 2,913 undergraduate college students from 30 U.S. universities in 19 states and the District of Columbia. Data were collected between April 21 and May 14, 2020, a period during which these universities had all transitioned to remote learning to avoid the spread of COVID-19 on campus. The number of students per university ranged from 1 to 1,257 with a mean of 97 (*SD* = 224) and a median of 46 students. A full table of the included institutions is available in S1 Appendix.

Given the unexpected nature of the pandemic and university closures, this study was launched promptly and used a convenience sampling frame. While a pre-planned project could produce a more representative sample of U.S. universities and their students (e.g., through stratified sampling), this work has the strategic advantage of capturing a large-scale, emergent phenomenon in real time, when a more complex sampling strategy would have been unfeasible. Participants were recruited by contacting university instructors at our own institution and via the authors’ professional networks at other universities nationally and asking them to distribute the survey link to students in their own classes and departments, and to colleagues across their universities.

The study and the survey protocol were approved by the Rutgers University Institutional Review Board (protocol # 2020000881) and the data were collected through an online questionnaire using the Qualtrics platform. Participants clicked the “I agree” option to consent to participate. All participants had to indicate that their university had transitioned to remote learning for the rest of their spring 2020 term before they took the survey and that they were age 18 or older. The survey introduction stated that the goal was “to understand students’ experiences with universities’ shifts to remote learning environments due to the COVID-19 pandemic,” and indicated that participation would take 10 minutes and be anonymous.

### Sample demographics

Our sample was demographically diverse. Students’ ages ranged from 18 to 40, with 94% of respondents being between ages 18 and 24. The participants were 65% female, somewhat more skewed than the general 4-year college population (56% female and 44% male), according to the National Center for Education Statistics [27]. Ten percent of participants were African American, 18% were Asian, 13% were Hispanic, and 62% were White. The numbers sum to over 100% because respondents could select more than one race or ethnicity. Overall, 17% of respondents selected multiple races or ethnicities.

According to U.S. Census Bureau data from the American Community Survey (ACS) in 2018, 16% of U.S. college students were African American, 8% were Asian, 20% were Hispanic, and 69% were White (54% non-Hispanic White). Differences from our survey sample likely result from the geographic distribution of the included universities. Our sample also differs from ACS data as we only included four-year colleges, and the ACS also includes two-year undergraduate institutions. In addition, 7% of our sample were international students, who reported being permanent residents of countries outside the U.S.; 72% were U.S. residents attending college in-state, and 21% out-of-state. Twenty-two percent of the sample were first-generation college students, and 9% were attending private universities.

### Measures

#### Endogenous variables

*Remote learning proficiency (RLP)* was measured by asking participants how much they agreed with three statements on a Likert-type scale ranging from 1 (*Strongly disagree*) to 5 (*Strongly agree*). We asked students whether, in the remote learning environment, they had trouble (1) understanding what instructors expected of them; (2) keeping track of deadlines and due dates; and (3) figuring out how to use programs they needed for their coursework (e.g., Zoom, Google Classroom). The items were reverse-coded so that a higher score meant having fewer issues navigating their remote learning environments. The scale had satisfactory internal consistency (α = .73). The final *remote learning proficiency* (RLP) measure (range 1–5, M = 2.7, SD = 1.1) was calculated by averaging the reverse-coded items.

*Communication challenges* were measured by asking students how much they agreed with four interrelated statements on a Likert-type scale ranging from 1 (*Strongly disagree*) to 5 (*Strongly agree*). The items asked students about (1) not being to communicate with professors as much as they would like; (2) not being able to communicate with teaching assistants as much as they would like; (3) finding it easier to connect with or relate to professors; and (4) finding it easier to connect with or relate to teaching assistants. The last two items were reverse-coded to create a measure where a higher score meant having more challenges in connecting with instructors. The scale had acceptable internal consistency (α = .72). A confirmatory factor analysis also pointed to an acceptable fit (*N* = 2845; *χ*^2^ = 12.7, *p* = .002, *DF* = 1; *RMSEA* = .06; *SRMR* < 0.01; *TLI* = .98; *CFI* = 1). The final measure (range = 1–5, *M* = 3.5, *SD* = 0.9) was calculated by averaging all items.

Consistent with our goal of exploring multiple dimensions of first-level digital inequality, we separately examined connectivity and device challenges during remote learning within the model. We also assessed experiences of being under-connected in the year prior to remote learning, consistent with past research showing that being under-connected is a condition that varies over time.

*Connectivity challenges* were measured by asking participants how much they agreed with four statements on a Likert-type scale ranging from 1 (*Strongly disagree*) to 5 (*Strongly agree*). The statements included (1) having a slow internet connection; (2) not being able to reliably livestream video; (3) having no reliable access to recorded lectures; and (4) not being able to download large files. The scale had good internal consistency (α = .78). A confirmatory factor analysis found an acceptable fit (*N* = 2848; *χ*^2^ = 67.5, *p* < .001, *DF* = 1; *RMSEA* = .1; *SRMR* = .3; *TLI* = .92; *CFI* = .97). The final measure (range 1–5, *M* = 2.4, *SD* = 1.0) was calculated by averaging the four items.

*Device challenges* were evaluated by asking respondents how much they agreed with three statements on a Likert-type scale from 1 (*Strongly disagree*) to 5 (*Strongly agree*). The statements included (1) having to share devices with too many other people; (2) using devices that were slow or in poor working condition; and (3) having to mostly use a smartphone to complete schoolwork. The scale had satisfactory internal consistency (α = .73). The final measure (range 1–5, M = 2.0, SD = 1.0) was calculated by averaging the three items.

Students’ experiences of being *under-connected in the prior year* were assessed via three validated measures of how students could have experienced these challenges in the past 12 months: having their internet service cut off due to inability to pay (7%); having reached the cap on their mobile data plan before the end of the month (28%); and having their laptop broken for 10 or more non-consecutive days (18%). Students were asked to select the statements that applied to them. The final measure (range 0–3, *M* = 0.5, *SD* = 0.7) was calculated by summing the three items.

#### Exogenous variables

The exogenous variables in our model included demographic controls for gender, race/ethnicity, year in school (from freshman to senior), and first-generation student status. We also controlled for whether the participant was an international student, an in-state student, or an out-of-state student as a proxy for how geographically disruptive campus shutdowns may have been for students. We controlled for whether a participant went to a private university, where the majority of the student body may be less likely to experience digital inequality challenges due to higher median family incomes, as compared to public universities.

Two variables were included to account for the financial situation of participants and their families in the early weeks of the pandemic, given that digital inequality and economic circumstances are closely related. *Financial hardship* asked students whether their own financial situation had taken a turn for the worse since the start of the pandemic. Responses were measured on a scale ranging from 1 (*Strongly disagree*) to 5 (*Strongly agree*) with *M* = 3.0 and *SD* = 1.4.

*Family economic insecurity* was a dichotomous variable, 1 for participants who reported having financially insecure families (25%) and 0 otherwise (75%). While this is a less precise measure than total household income, prior studies indicate that only one-quarter of students can accurately predict their parents’ income within $15,000 [28]. We therefore opted for a simpler and more emic measure of economic (in)security, in a period where many families’ financial situations had potentially become more precarious.

The number of *past online classes* that students had taken in previous semesters (range 0–11, *M* = 2.0, *SD* = 2.8) was also included, as extant research indicates that previous experience with online learning might have enabled students to cope better with the transition to remote classes. Forty-one percent of survey participants had never taken an online course prior to remote learning, and another 16% had only taken a single online class previously.

## Analysis and results

Hypothesis testing was conducted using a structural equation model with cluster-bootstrapped standard errors and p-values to account for the fact that the students were clustered by university. The analyses were conducted using the R platform for statistical computing version 4.0 and RStudio version 1.3.1056, along with *lavaan* package [29]. The hypothesized structural model is presented in Fig 1. To determine whether the hypotheses were supported, we examined the significance of individual paths and the global fit of the model to the observed data. A total of 103 cases (3.5% of the data) had missing values for the model variables due to item non-response and were thus excluded from the analysis. To deal with missing data in the main analysis, we use full information maximum likelihood (FIML) estimation.

**Fig 2:**
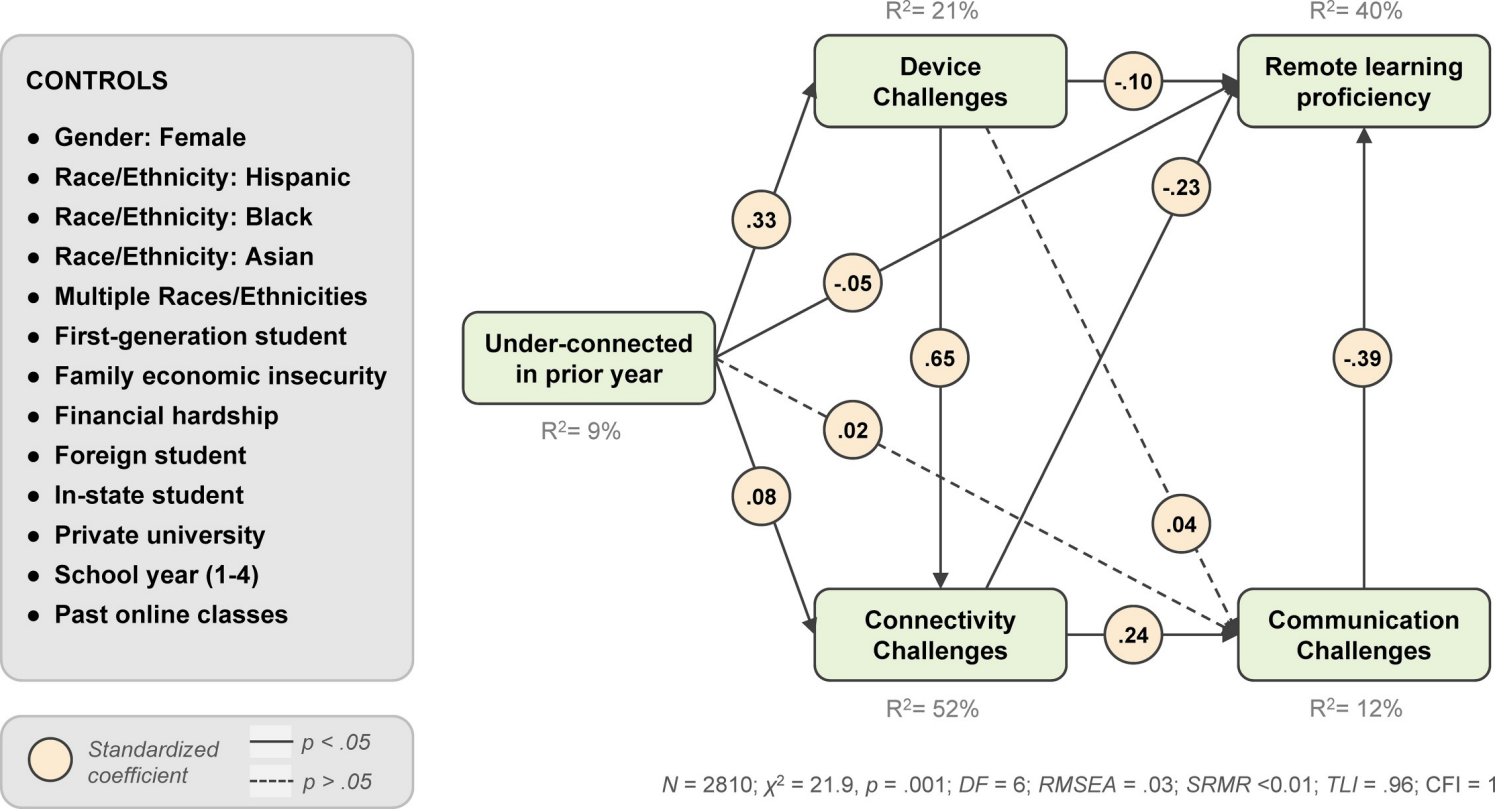
Structural equation model for remote learning challenges and proficiency. The figure includes standardized coefficients.

Overall, the model had a good fit (*N* = 2810; *χ*^2^ = 21.9, *p* = .001, *DF* = 6; *RMSEA* = .03; *SRMR* < 0.01; *TLI* = .96; *CFI* = 1). All paths among endogenous model variables (with the exception of the path from being under-connected in the prior year to communication challenges) were significant at *p* < .05. The full model results are presented in Table 1 and Fig 2.

**Table 1:** Structural equation model results, standardized coefficients with clustered standard errors included in parentheses.

Variable	Remote learning proficiency	Communication challenges	Connectivity challenges	Device challenges	Under-connected in prior year
Communication challenges	-.39 (.02)***	-	-	-	**-**
Connectivity challenges	-.23 (.03)***	.24 (.03)***	-	-	-
Device challenges	-.10 (.02)***	.04 (.02)	.65 (.01)***	-	-
Under-connected in prior year	-.05 (.01)***	.02 (.02)	.08 (.02)***	.33 (.02)***	-
Gender: Female	-.02 (.02)	.03 (.02)	.09 (.01)***	-.06 (.02)*	-.03 (.01)**
Race/Ethnicity: Hispanic	-.03 (.02)	-.03 (.03)	-.03 (.01)*	.01 (.03)	-.00 (.02)
Race/Ethnicity: Black	-.02 (.01).	-.04 (.01)*	-.06 (.01)***	.02 (.02)	.07 (.02)**
Race/Ethnicity: Asian	.00 (.01)	-.02 (.01)	-.02 (.01).	.01 (.02)	-.06 (.02)***
Multiple Races/Ethnicities	-.02 (.02)	.04 (.03)	.04 (.02)*	.01 (.03)	.04 (.02)
Family economic insecurity	.01 (.01)	.03 (.02)	-.02 (.01).	.05 (.01)***	.13 (.02)***
Financial hardship	-.12 (.02)***	.06 (.02)***	.09 (.01)***	.19 (.02)***	.21 (.02)***
First-generation student	-.01 (.01)	-.02 (.01)*	.00 (.01)	.02 (.02)	-.03 (.02)
Foreign student	.05 (.01)***	-.12 (.01)***	-.05 (.01)***	.04 (.02)*	.04 (.02)**
In-state student	-.01 (.01)	-.02 (.02)	.00 (.02)	.01 (.03)	.01 (.02)
Private university	.02 (.03)	-.02 (.02)	.02 (.02)	-.00 (.01)	-.02 (.02)
Past online classes	.04 (.01)**	-.07 (.02)***	-	-	-
School year (1–4)	.02 (.02)	-.03 (.02).	-	-	-
R^2^	40%	12%	52%	21%	9%

p < .1

* p < .05

** p < .01

*** p < .001

H1 and H2 suggested that *connectivity challenges* and *device challenges* would be associated with lower *remote learning proficiency*. Our results support both hypotheses: RLP was significantly negatively related to both connectivity challenges (*β* = -.23, *SE* = .03, *p* < .001) and device challenges (*β* = -.10, *SE* = .02, *p* < .001).

H3 predicted that *communication challenges* would be associated with lower *RLP*. Indeed, difficulty communicating with professors and teaching assistants was the largest in magnitude negative predictor of RLP (*β* = -.39, *SE* = .02, *p* < .001).

RQ1 asked about associations between previous technology experiences and RLP. In the structural equation model, being *under-connected in the prior year* had a relatively small, negative direct effect on *RLP* (*β* = -.05, *SE* = .01, *p* < .001). It also had small one-step indirect effects through its links to *device*, *connectivity*, and *communication challenges* (*β* = -.06, *SE* = .03, *p* < .01). The most important role that being *under-connected in the prior year* played in the model was as a predictor of the *device challenges* students reported during remote learning in the spring term (*β* = .33, *SE* = .02, *p* < .001).

Having taken *past online classes* also had a small, but significant, positive association with *RLP* (*β* = .04, *SE* = .01, *p* < .01), and a significant, negative association with c*ommunication challenges* (*β* = -.07, *SE* = .02, *p* < .001).

Finally, RQ2 asked whether students’ assessments of their own and their families’ current financial insecurity were associated with their RLP, as well as challenges with connectivity, devices, and faculty communication. Students’ reports of personal *financial hardships* resulting from the pandemic was significantly and negatively associated with *RLP* (*β* = -.12, *SE* = .02, *p* < .001), and significantly, positively associated with *device challenges* (*β* = .19, *SE* = .02, *p* < .001), *connectivity challenges* (*β* = .09, *SE* = .01, *p* < .001), and *communication challenges* (*β* = .06, *SE* = .02, *p* < .001). *Family economic insecurity* was significantly associated with having been *under-connected in the prior year* (*β* = .13, *SE* = .02, *p* < .001) and with *device challenges* (*β* = .05, *SE* = .01, *p* < .001).

## Discussion

The onset of the COVID-19 pandemic in the U.S. prompted a shift in undergraduates’ college experiences that would have been unimaginable only months earlier. The rapid pivot to remote learning required mastering new learning platforms and forms of communication, as well as a sudden, complete dependence on a digital device and steady internet connection. For many, this rapid learning shift also entailed moving back into their family home [30]. In all this upheaval, what factors contributed to whether students developed *remote learning proficiency*: that is, the ability to successfully navigate this new, unfamiliar learning environment?

While all students faced the challenges associated with adapting to novel conditions in the middle of a semester, they did not begin that hasty transition on equal ground. Prior research suggested, and our own findings confirm, that structural differences based on students’ economic dis/advantages and prior challenges in securing adequate internet and functioning digital devices were associated with students’ sense of remote learning proficiency (RLP). The pandemic put existing structural inequities into stark relief, in addition to creating new challenges due to the unplanned, mid-term transition.

Our first two hypotheses predicted that students with more connectivity and device challenges would have lower RLP. It is not surprising that students with unstable broadband internet and whose digital devices need technological maintenance had more trouble managing online course platforms, tracking due dates, and knowing what is expected from them in remote learning environments. Furthermore, extant research indicates that under-connected students are disproportionately likely to be in positions of financial hardship, and therefore, to displace crucial time and energy from their coursework as they develop workarounds for spotty internet and malfunctioning devices even when classes are face-to-face [9]. These challenges are, obviously, amplified when the internet and digital devices become the sole means for engaging in coursework.

Perhaps a more surprising finding from our analyses is that connectivity and device challenges explain less of the variance in RLP than do communication challenges. Our findings support Hypothesis 3: students who experience challenges communicating with their professors and teaching assistants have lower RLP. It may be that students’ casual opportunities to chat with instructors before or after class, or by passing by their instructors’ offices, could not be adequately replicated in remote instruction. Consistent with prior, smaller studies, our findings suggest that instructors play a pivotal role in whether students develop RLP, depending on whether they communicate clearly and responsively with students and maintain clear expectations, predictable deadlines, and consistent learning formats on online platforms [24].

Our first RQ asked about associations between RLP and two specific types of prior technology experiences: namely, having taken online courses prior to the pandemic, and having been under-connected in the prior 12 months.

As might be expected, having completed past online classes had a significant, positive association with RLP. This finding potentially reflects a selection bias, since these participants had voluntarily opted for an online learning experience when the choice of face-to-face instruction was available instead. Furthermore, their prior experience with online learning accorded them greater familiarity with the platforms and expectations that came to be associated with remote learning, compared with classmates who had neither. Students with prior online learning experience were also less likely to report communication challenges with their instructors. This suggests either an affinity for the forms of communication that technology can foster, or at least, a familiarity with the affordances and limitations of the communication channels available for interaction with instructors online.

Our measures also identified students who had experienced one or more of the following experiences of being under-connected in the prior year: having their internet service cut off due to inability to pay; hitting the cap on their mobile data plan mid-month; or having a broken laptop for 10 or more non-consecutive days, all of which have been validated as key aspects of being under-connected in prior digital inequality research [3, 9]. Having been under-connected in the prior year had a relatively small, significant association with both connectivity and communication challenges during remote learning, as well as small direct and indirect effects on RLP.

The most important role having been under-connected in the past year played in the model was as a predictor of the device challenges that students reported during remote learning. This finding is consistent with prior research on how technological maintenance issues can chronically bedevil lower-income populations [8]. It also underscores the importance of capturing under-connectedness across multiple time points, even in a cross-sectional survey. The ‘dependable instability’ [8, 9] of being under-connected means that a snapshot measure will not adequately capture which undergraduates struggle to maintain adequate connectivity and devices, since students who entered remote learning disadvantaged by being under-connected in the prior year continued to encounter problems once courses moved to remote instruction.

Our second RQ asked whether students’ assessments of their own and their families’ current financial security was associated with remote learning challenges and proficiency. Students’ economic circumstances in the early days of the pandemic had important associations with RLP. Specifically, reporting increased financial hardships since the onset of the pandemic was significantly, negatively related to RLP, and positively associated with connectivity, device, and communication challenges. Similarly, reports of familial economic insecurity were significantly, positively associated having been under-connected in the past year and with device challenges during remote instruction. These findings underscore how many students experienced remote learning challenges early in the pandemic as part of a much broader set of insecurities.

### Limitations

The study has some limitations that are important to keep in mind. The first is a corollary of one of the dataset’s strengths: that we captured students’ remote learning experiences in real time. The limitation is that students may have reported their RLP differently once the spring term was over and they had had some time to reflect on the totality of their experience.

The second limitation is that the generalizability of the findings is constrained by the convenience sampling method employed to gather the data. We contend that this limitation is offset, to some degree, by the size of the sample and its diversity in terms of the number and geographic distribution of universities represented, as well as the racial/ethnic and economic diversity of the students who participated. Furthermore, given our desire to capture students’ experiences before the term ended and with the constraints of pandemic teaching in place, a more rigorous sampling strategy was not feasible at the time that these data were collected. As studies of remote learning continue in relatively more stable conditions going forward, it will be important to prioritize strategies for more representative sampling.

Third, our survey questions were informed by our own and others’ prior research on digital inequality and learning experiences but were adapted to our goal of understanding remote learning as it was unfolding on its own unique terms. Though the measures we developed were, we believe, well-informed, they were also necessarily exploratory. Follow-up research is needed to extend and fully validate those measures, as well as to examine measurement invariance. Additionally, future studies should explore not only student characteristics, but also instructor and course attributes. That should help to better explain variables which are more heavily dependent on these factors, such as the *communication challenges* students experienced.

Finally, we acknowledge the potential for two forms of respondent bias in the sample. It is possible that students experiencing severe under-connectedness would have been less likely to take the survey at all. We did our best to mitigate this possibility by optimizing the survey for mobile devices and by keeping the survey to 10 minutes. And given the pandemic conditions, we were most likely to successfully recruit participants via faculty we knew, at least indirectly, as opposed to emailing strangers during a stressful time. This created the unavoidable reality that many of the colleagues we contacted nationally are in our own or related fields. We attempted to mitigate this imbalance by drawing on our colleagues’ networks, asking them to send the survey on to faculty they know in other departments.

## Conclusions

Our results suggest that there are two kinds of connection that students need to develop remote learning proficiency: digital connectivity, in the form of consistent, high-speed internet and functional digital devices on the one hand, and strong human connections to the instructors who guide their learning, on the other. While the former provides the foundational infrastructure for students’ access to a novel learning environment, the latter provides the supportive framework to develop the digital skills to successfully navigate it, as well as the motivation to persist until that proficiency is realized.

As such, this study contributes to digital inequality research by identifying how first- and second-level digital inequality are connected within the sudden shift to remote learning during the early stages of the pandemic. Our findings are also consistent with extant literature in finding that financially insecure students report more challenges to maintaining the internet connectivity and devices that enable consistent access to remote learning environments. However, under-connected students may be even more vulnerable in remote than in face-to-face learning conditions, given that digital access is also prerequisite for communicating and securing assistance from teaching assistants and professors in remote learning.

While remote learning will continue in some form until the pandemic resolves, the reverberations of this unprecedented period of undergraduate education will be felt for much longer. Our study reveals some of the key factors associated with students’ confidence that they can successfully navigate an unfamiliar learning environment. The longer-term question is how developing RLP will relate to the kinds of outcomes that third-level digital inequality research has focused on, including college graduation rates. In extreme cases, students who feel that they cannot succeed in remote learning may drop out of college entirely. Equally troubling, however, is the larger number of students who will remain in college, but struggle to be fully engaged in learning valuable material, developing new skills, and interacting meaningfully with instructors and fellow students, due to digital inequality.

To fully capture how first- and second-level digital inequality are influencing undergraduates’ outcomes from remote learning will require longitudinal studies, Over time, it will be possible to trace how the volatility and vulnerability of being under-connected affects accessing course content and communicating with instructors. Our study’s contribution to the nascent and urgent effort to understand this unintended national experiment in undergraduate education is in providing a clear snapshot of how students experienced the very earliest weeks of the remote learning transition, and of what supported the very earliest stages of their adaptation.

## Supporting information

S1 Appendix (DOCX)
