# Supplementary material for: Digital inequality, faculty communication, and remote learning experiences during the COVID-19 pandemic: A survey of U.S. undergraduates
Source: PLoS One. 2021 Feb 10;16(2):e0246641. doi: 10.1371/journal.pone.0246641 (PMC7875367; doi:10.1371/journal.pone.0246641)
Supplement: S1 Appendix — (DOCX) [file pone.0246641.s001.docx]

**Appendix: Additional Tables**

Table A1. Universities included in the data sample

| # | **University** | **State** | **N Students** |
| --- | --- | --- | --- |
| 1 | Drexel University | Pennsylvania | 14 |
| 2 | Georgetown University | District of Columbia | 42 |
| 3 | Georgia State University | Georgia | 95 |
| 4 | Michigan State University | Michigan | 202 |
| 5 | Mississippi State University | Mississippi | 55 |
| 6 | Muhlenberg College | Pennsylvania | 34 |
| 7 | Northeastern University | Massachusetts | 6 |
| 8 | Northwestern University | Illinois | 6 |
| 9 | Oklahoma State University | Oklahoma | 166 |
| 10 | Penn State University | Pennsylvania | 122 |
| 11 | Rutgers University | New Jersey | 1257 |
| 12 | Temple University | Pennsylvania | 115 |
| 13 | University of Alaska - Fairbanks | Alaska | 20 |
| 14 | University of Arizona | Arizona | 27 |
| 15 | University of California - Irvine | California | 17 |
| 16 | University of California - San Diego | California | 1 |
| 17 | University of California - Davis | California | 20 |
| 18 | University of California - Santa Barbara | California | 51 |
| 19 | University of Cincinnati | Ohio | 42 |
| 20 | University of Connecticut | Connecticut | 59 |
| 21 | University of Delaware | Delaware | 90 |
| 22 | University of Denver | Colorado | 47 |
| 23 | University of Illinois | Illinois | 3 |
| 24 | University of Maryland | Maryland | 42 |
| 25 | University of Michigan | Michigan | 14 |
| 26 | University of Minnesota | Minnesota | 101 |
| 27 | University of San Diego | California | 44 |
| 28 | University of Southern California | California | 62 |
| 29 | University of Texas - Austin | Texas | 99 |
| 30 | University of Washington | Washington | 60 |
